# First description of a sporadic breast cancer in a woman with *BRCA1* germline mutation

**DOI:** 10.18632/oncotarget.5348

**Published:** 2015-09-29

**Authors:** Elsa Curtit, Vanessa Benhamo, Nadège Gruel, Tatiana Popova, Elodie Manie, Paul Cottu, Odette Mariani, Dominique Stoppa-Lyonnet, Xavier Pivot, Marc-Henri Stern, Anne Vincent-Salomon

**Affiliations:** ^1^ Department of Medical Oncology, Institut Curie, 75248 Paris, France; ^2^ Department of Medical Oncology, University Hospital, 25000 Besançon, France; ^3^ University of Franche-Comté, Medical Department, 25000 Besançon, France; ^4^ INSERM U1098, Medical Oncology Department, 25000 Besançon, France; ^5^ Université Paris Sciences Lettres, Medical Department, INSERM U934, Institut Curie, 75248 Paris, France; ^6^ Université Paris Sciences Lettres, Medical Department, INSERM U830, Institut Curie, 75248 Paris, France; ^7^ Department of Translational Research, Institut Curie, 75248 Paris, France; ^8^ Department of Pathology, Genetics and Immunology, Institut Curie, 75248 Paris, France; ^9^ Present affiliations: 2–4; affiliation when working on this case: 1

**Keywords:** breast cancer, BRCA1, HER2, sequencing

## Abstract

We describe the case of a woman carrying a germline pathogenic *BRCA1* mutation diagnosed with a breast cancer overexpressing HER2. Clinical presentation of the tumor, HER2-positivity, genomic profile and loss of the mutated *BRCA1* allele in tumor evidence that BRCA1 is not inactivated in this breast cancer. It represents the first biological demonstration for the existence of a sporadic HER2-positive breast cancer independent from BRCA loss of function in a woman carrier of a deleterious *BRCA1* mutation. In a context where targeted therapies based on BRCA loss of function in the tumor are developed, such case could have direct implications.

## INTRODUCTION

Microarray-based expression profiling studies have refined our comprehension of breast cancer biology and have triggered a paradigm in breast cancer taxonomy leading to the classification of breast cancers in at least five intrinsic subgroups [1]. HER2-positive breast cancers and basal-like breast cancers constitute two of these subgroups.

Basal-like breast carcinomas belong to the triple-negative (TN) breast cancers family, with no expression of estrogen receptor (ER), progesterone receptor (PR) and no HER2 overexpression (amplification). Initially, basal-like breast cancers were named after the expression of genes found in the myoepithelial / basal cells, located at the basal part of the mammary gland of the human breast, such as cytokeratins 5, 6, 14, 17 and an absence of *ESR1* gene expression (ER negative) [2].

**Figure 1:**
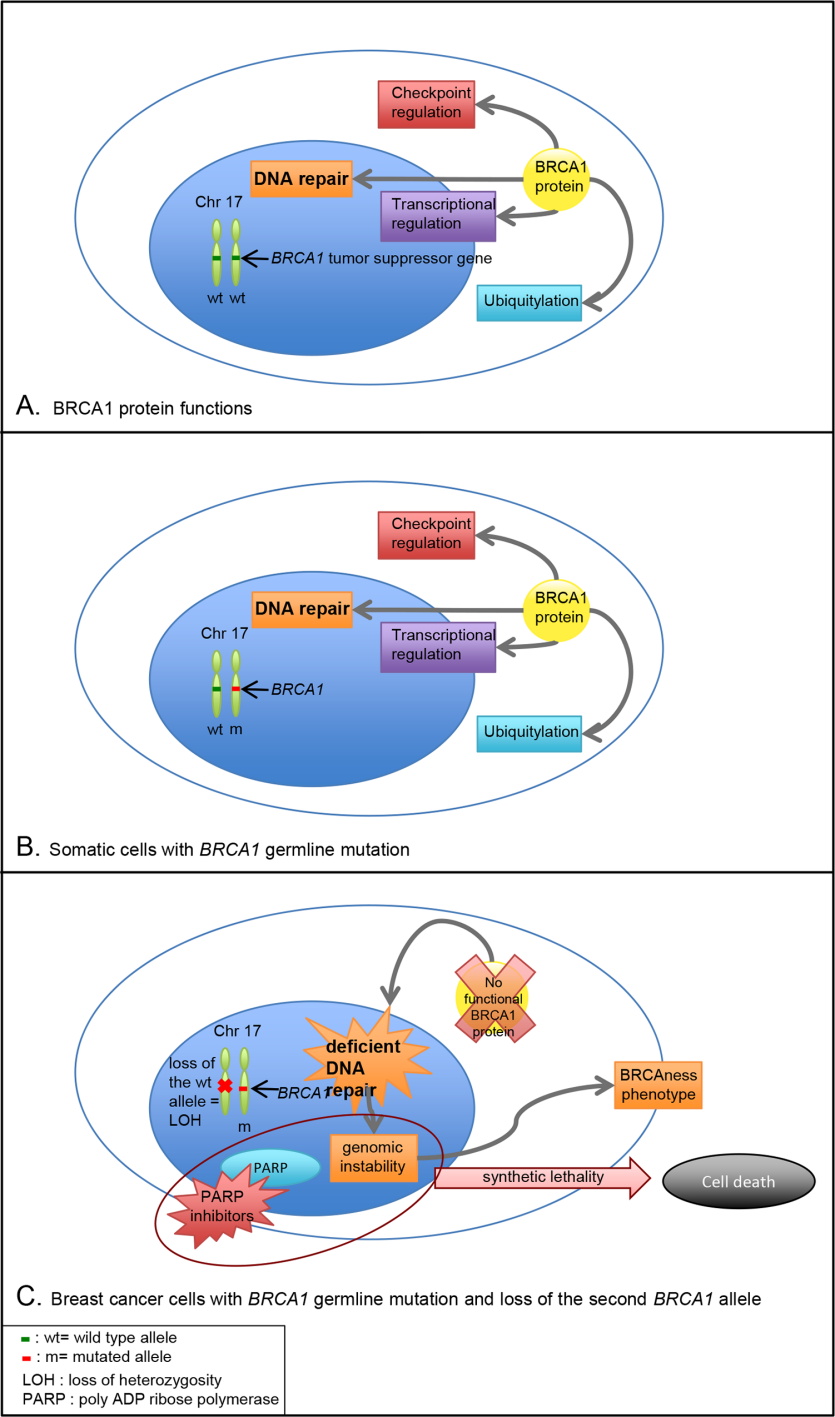
BRCA1 function and consequences of *BRCA1* germline mutation in somatic cells and breast cancer cells **A.** *BRCA1* tumor suppressor gene encodes a protein with diverse biological functions playing pivotal roles in DNA repair, cell-cycle checkpoints, ubiquitylation and transcriptional regulation. **B.** In patients with *BRCA1* germline mutation, one allele of the gene is mutated and the *second wild type allele encodes a BRCA1 functional protein. Germline biallelic mutations in BRCA1* are responsible for Fanconi anemia. **C.** In breast cancer cells with *BRCA1* germline mutation, one allele of the gene is mutated and the other is lost (commonly by a deletion), which is responsible for a loss of heterozygosity. The lack of BRCA1 functional protein leads to deficient DNA repair and genomic instability conferring a BRCAness phenotype. The inhibition of PARP in these cells, based on the concept of synthetic lethality, majors genomic instability and leads to cell death.

Germline mutations in the tumor suppressor gene *BRCA1* (17q21) are one of the main known causes of hereditary early onset breast and ovarian cancer syndrome. Germline mutations *BRCA1* and *BRCA2* explain around 20% of familial breast cancer [3–5]. Other rare variants in high penetrance genes such as *PALB2, CHEK2, ATM, NBN, TP53, CDH1, PTEN, STK11* and *NF1* [6] confer moderate or high risk of developing breast cancer. *BRCA1* gene acts as tumor suppressor and plays a role in maintenance of genomic stability through DNA damage recognition and repair (Figure 1). *BRCA1* mutations are inherited in an autosomal dominant fashion with variable penetrance. *BRCA1*-related breast cancers follow the “two-hits hypothesis”: it implies that both *BRCA-1* alleles are altered in the tumor. The “1^st^ hit” is constituted by the germline mutation, which alters one allele of the *BRCA1* gene. In familial *BRCA* cancers, the constitutional mutation is the first event and brings a loss of heterozygosity. The “2^nd^ hit” appears in tumor cells with the loss of the second allele, commonly by a deletion. Thus, there is a loss of the expression of the BRCA1 wild type protein. *BRCA1* encodes a protein with diverse biological functions playing pivotal roles in DNA repair (especially by homologous recombination), cell-cycle checkpoints, ubiquitylation and transcriptional regulation [7, 8]. Homologous recombination deficiency leads to defective double-strand break repair and genomic instability. A specific phenotype reflecting double-strand break repair pathway deficiency was described [7, 9] both in familial and in sporadic breast cancers. PARP inhibitors are new anti-cancer agents, which inhibit the enzyme poly ADP ribose polymerase involved in repairing single-strand breaks. The use of PARP inhibition in tumors with BRCA deficiency, based on the concept of synthetic lethality, induces cell death through the accumulation of DNA alterations in cells with multiple DNA repair deficits [10]. Contrary to non-hereditary breast cancers, tumors arising in women carrying a germline *BRCA1* mutation present preferentially with a basal-like subtype and thus in most cases a triple negative phenotype in ∼85% of the cases [11–13].

HER2-positive breast cancers are mostly characterized by an amplification of the *HER2* gene (human epidermal growth factor receptor 2, located at 17q12) associated with gene overexpression and consequently high abundance of HER2 protein. HER2 is involved in growth, differentiation, and cell survival. Patients with HER2-positive tumors have a spontaneous poorer prognosis than other subtypes with significantly shortened disease-free survival and overall survival [14]. The advent of trastuzumab, a humanized monoclonal antibody targeting specifically the HER2 extracellular domain, has revolutionized the natural history and management of HER2-positive breast cancers [14–16].

The data describing HER2-positive breast cancers occurring in women carrying a germline *BRCA* mutation are rather poor and evidence rarity of such phenotype [17–22]. We report here the case of a 40 year-old woman carrying a pathogenic germline *BRCA1* mutation and diagnosed with a HER2-positive breast cancer. Two scenarii might be possible to explain the case: (1) it is sporadic HER2-positive breast cancer developed in a patient with *BRCA1* germline mutation and not causally linked to the tumor suppressor gene *BRCA1* loss-of-function and in such case we could introduce the notion of incidental genomic event, or (2) it is a rare subgroup of breast cancers occurring in patients carrying *BRCA1* germline mutation in which *BRCA1* loss of function and HER2 overexpression cooperate in the oncogenesis of the tumor. A series of molecular analyses including immunohistochemistry, fluorescence *in situ* hybridization (FISH), sequencing and SNP-array profiling were performed to characterize this tumor.

### Presentation of the case

A 40-year-old premenopausal woman was referred to *Institut Curie* Medical Oncology Department with a newly diagnosed breast carcinoma. Approximately 2 months earlier, she had noted a lump in her right breast. Digital mammography showed heterogeneously dense breast parenchyma with no mass, architectural distortion or suspicious microcalcifications. Ultrasonography of the breast confirmed the presence of a solid hypoechogenic mass in the right breast, with irregular margins, measuring 20 × 15 mm. A core biopsy was performed and revealed a poorly differentiated invasive ductal carcinoma.

On initial physical examination, breasts were symmetric and soft. A 20 mm nodule was palpated in the right lower outer quadrant. The nipples and skin were normal, with no erosions or inflammatory lesions. No palpable lymph nodes were found. The left breast and the remainder of the examination were unremarkable. The results of routine hematologic and blood chemical tests were normal. A computed tomography of the chest, abdomen and pelvis and an isotopic bone scan showed no evidence of metastatic lesions.

The patient had no other medical condition and her medical history was restricted to an appendectomy at 16-year-old. Her mother and her maternal grandmother had ovarian cancer at the age of 50 and 57 respectively.

**Figure 2:**
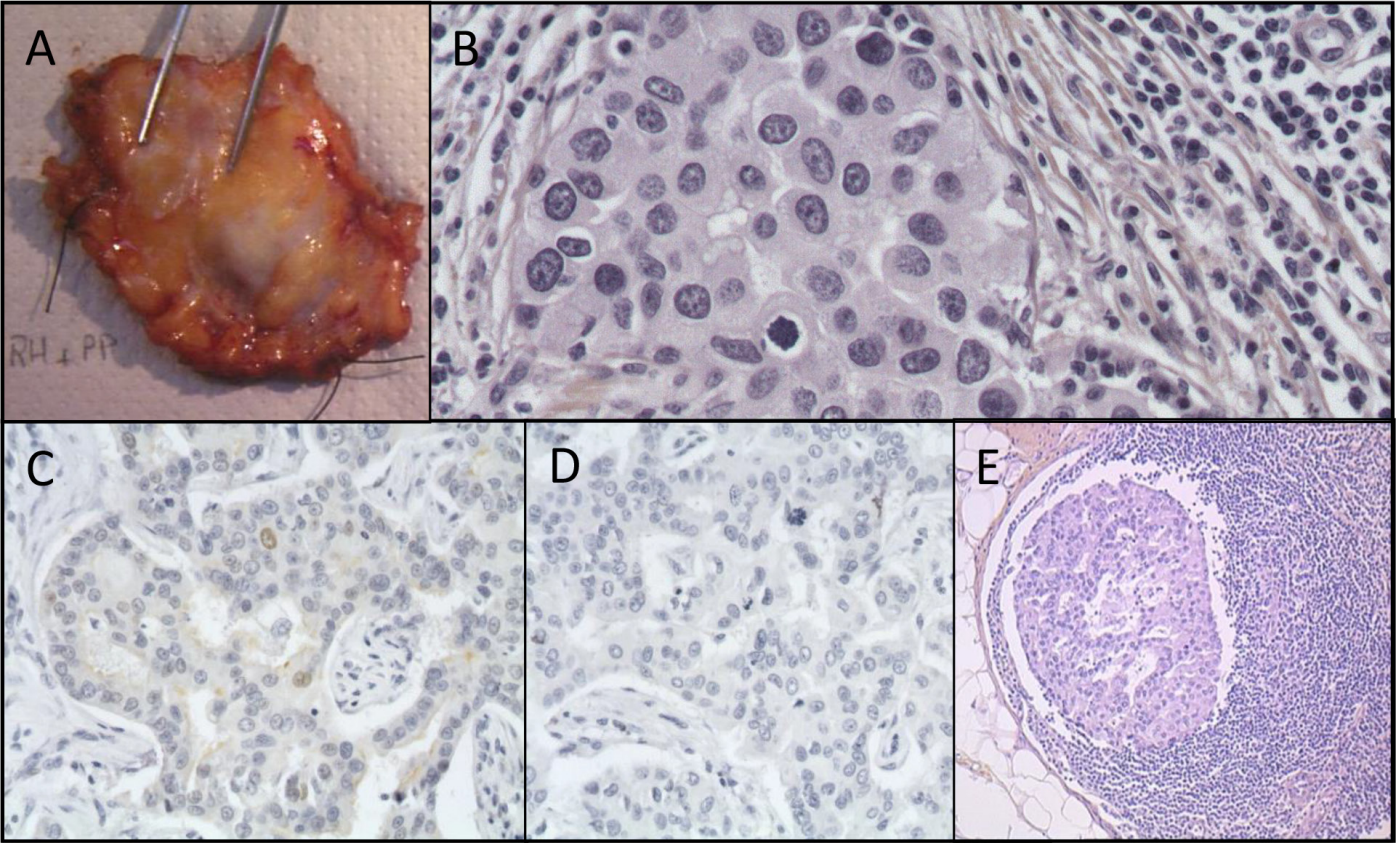
Histopathological features of the breast excision specimen **A.** Gross aspect of the breast surgical excision specimen **B.** Hematein-eosin saffron staining from the primary breast tumor **C.** Estrogen receptor immunostaining of the primary tumor. Less than 10% of positive nuclei **D.** Progesterone receptor immunostaining of the primary tumor. No positive nuclei **E.** Haematoxylin-eosin-saffron staining tissue section from axillary lymph node metastasis.

**Figure 3:**
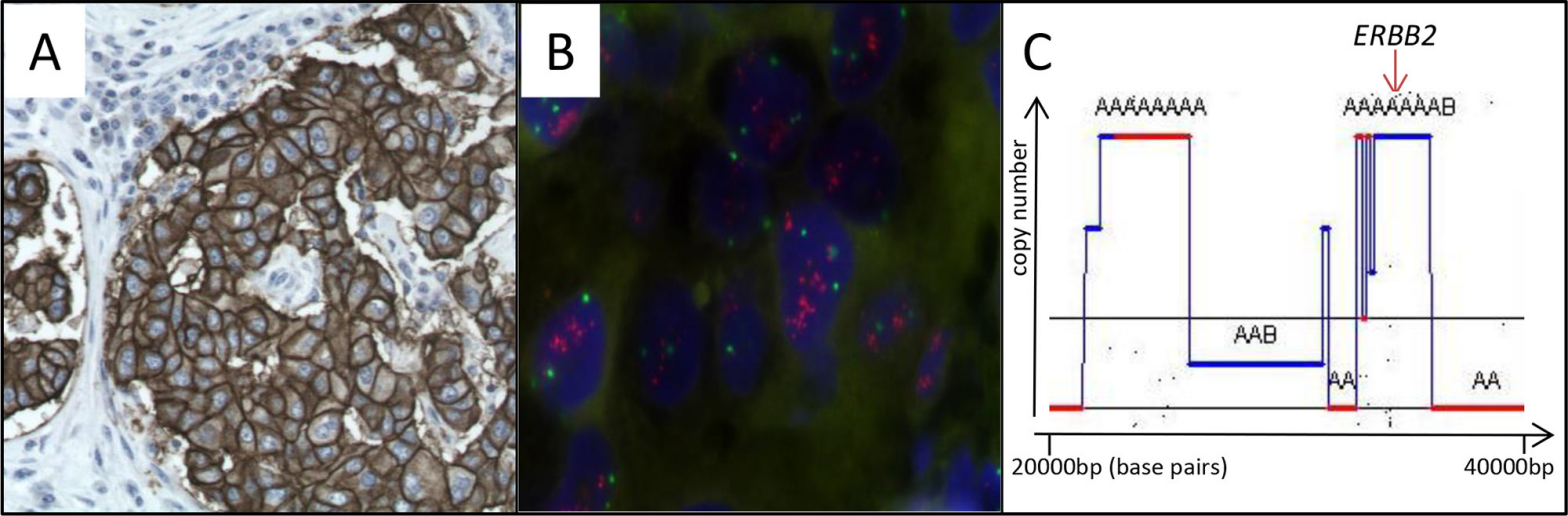
Confirmation of *ERBB2* / HER2 amplification and overexpression **A.** Strong immunostaining (CB11 antibody) of HER2 membrane protein **B.** FISH with *ERBB2* probe on a tissue section of the invasive breast carcinoma. Ratio *ERBB2*/chromosome 17 centromeres = 3.3, confirming *ERBB2* amplification **C.** SNP6.0 analysis with GAP ^24^ focused on the chromosome 17q1.2 locus showing *ERBB2* amplification.

The patient underwent breast-conserving surgery of the right breast (Figure 2A) with concomitant excision of 2 sentinel nodes and 10 non-sentinel axillary nodes. Microscopic analysis of the tumor revealed an infiltrating ductal carcinoma poorly differentiated (Figure 2B), with high mitotic index, 20 mm in greatest dimension, Elston and Ellis grade was scored at 3/3. Foci of ductal carcinoma *in situ* were present in the vicinity of the main mass. Surgical margins were clear. On immunohistochemical stainings, the tumor was negative for ER (Figure 2C) and PR (Figure 2D). The tumor showed strong overexpression of HER2 (3+) revealed by a membrane labeling on immunohistochemical staining (Figure 3A) [23]. CK5/6 and CK14 were not expressed. Lymphatic invasion was seen in 3 of 12 lymph nodes (Figure 2E) with tumor effraction of the capsule in one node.

According to our therapy guidelines, the patient received 6 cycles of adjuvant chemotherapy consisting of 3 cycles of 5-fluorouracil (500 mg/m^2^), epirubicin (100 mg/m^2^) and cyclophosphamide (500 mg/m^2^) and 3 cycles of docetaxel (100 mg/m^2^). Trastuzumab was started concomitantly with docetaxel at a loading dose of 8 mg/kg followed by 6 mg/kg every 3 weeks for one year. The patient received radiation therapy to the chest wall and regional lymph nodes, followed by an additional radiation boost to the mastectomy incision to a total dose of 60 Gy with a standard fractionation schedule.

**Figure 4:**
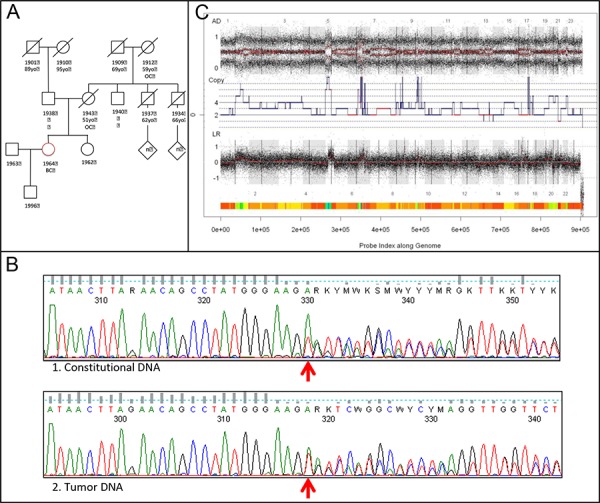
BRCA1 analyses and BRCAness **A.** Familial pedigree: Circles indicate female family members, squares male family members, slashes deceased family members, and the diamond multiple persons (exact number [n] unknown). Red circle indicates the proband. BC: breast cancer; OC: ovarian cancer; 19— : year of birth; —yo: age of death (year-old). **B.** Classic Sanger sequencing of *BRCA1* gene in the white blood cells (1) and in the tumor (2). At the tumor level the mutation in *BRCA1* was also detected (wild type allele (T) and mutated allele (A)), however, at the lower allelic proportion compared to the germline. Taking into account the 40–50% of tumor cells within the sample, it is compatible with the loss of *mutated* allele in the tumor sample. **C.** SNP6.0 analysis with GAP assessing BRCAness of the tumor.

The patient's young age and familial history of ovarian cancer (Figure 4A) triggered the testing for mutations in the *BRCA1* and *BRCA2* genes. She was found to carry a monoallelic c.3417delT;p.Ser1139ArgfsX16 *BRCA1* mutation (Figure 4B1) and no *BRCA2* mutation. The deletion alters the reading frame of the gene with occurrence of a codon, which encodes for a stop, 16 codons after the mutation (fsX16) and truncates the protein.

She opted for a prophylactic oophorectomy and chose to have breast surveillance follow up with regular mammography and magnetic resonance imaging. The oophorectomy sample material was without any pathologic signs. Eight years after the diagnosis of breast cancer, the patient is alive with no recurrence of the disease.

## RESULTS

### Confirmation of HER2 amplification and overexpression

FISH analysis highlighted the presence of 10 signals of the *HER2* (*ERBB2*) probe and three centromeres of chromosome 17 with a ratio *ERBB2*/centromeres = 3.3 (Figure 3B). These data confirmed that *HER2* was amplified in the tumor. SNP6.0 profile focused on *HER2* also concluded amplification of the locus with copy number >8 (copy number around centromere of chromosome 17 is 2) (Figure 3C).

### BRCA1 status

At the tumor level the mutation in *BRCA1* was also detected, however, at the lower allelic proportion compared to the germline (Figure 4B2). The BRCA1 gene was not in an amplified region. Copy number and allelic status of *BRCA1* locus inferred from SNP-arrays were 2 copies and loss of heterozygosity (LOH) (Supplementary Figure S1). Taking into account relatively low percentage of tumor cells within the sample (~40–50%) the sequencing data are compatible with the loss of *mutated* allele in the tumor sample.

### Tumor genomic profile analysis

Mining SNP-array tumor genomic profile by the GAP method allowed recognition of absolute copy number and allelic content profiles [24]. Normal contamination was estimated as ~50%; DNA index inferred from copy number profile was equal to 1.55, implying over-diploid or near-tetraploid DNA content (Figure 4C). According to several studies, the high level of genomic structural alterations is characteristic of *BRCA1* or *BRCA2* inactivation (so called BRCAness) in triple-negative breast and serous ovarian carcinomas [25–27]. We assessed BRCAness of a tumor based on the number of large-scale inter-chromosomal breakpoints (designated as Large-scale State Transitions, LSTs) in the annotated genomic profile. The number of LSTs in the tumor was found to be 16, which is lower than a cut-off for BRCAness (20 LSTs for tumors with DNA index ≥ 1.3) [27]. Thus tumor genomic profile evidences intact functioning of *BRCA1* and *BRCA2* and no homologous recombination defect.

### Other genes

*TP53* was not mutated (in hotspot exons 5 to 9). *PTEN* was also normal according to SNP-array profile (no gain, no loss and no LOH).

To conclude, clinical presentation of the tumor, proven HER2-positivity, absence of *TP53* mutation, genomic profile without characteristic pattern of BRCA1 inactivated tumors (no genomic BRCAness) and loss of the mutated *BRCA1* allele in tumor evidence that the case represented a HER2-positive breast cancer without BRCA1 inactivation.

## DISCUSSION

Well-established molecular subtyping of sporadic and hereditary breast tumors allowed consideration of rare cases with unusual genotype/phenotype co-occurrence. In the present case report we considered HER2-positive breast tumor occurred in the *BRCA1* germline mutated context. We addressed the question whether the patient with constitutive *BRCA1* mutation diagnosed with HER2-positive carcinoma at young age have developed a carcinoma driven by BRCA1.

What is interesting about the presented case is the existence of a LOH in *BRCA1* locus associated with the loss of the mutated allele in the tumor. At least 3 scenarii can be elaborated: 1/*BRCA1* mutation do not drive the oncogenesis of the tumor hence such a mutation would be considered as a “genetic incidentalom”. There would be no linkage between *BRCA1* mutation and the tumor and the tumor can be considered as a sporadic tumor. 2/*BRCA1* mutation triggers the initiation of the tumor but then does not control the tumor, this last been autonomous. Therefore it would be comparable to a “launch and forget” process. The lost of the mutated allele is fortuitous, due to genomic instability. 3/*BRCA1* mutation does initiate the tumor and the apparition of mutations triggers a cascade of reparatory mechanisms. The loss of the mutated allele illustrates an attempt of DNA repair process with no effect on the tumor development.

Besides the study of *BRCA1* alleles in the tumor, different patterns characterizing *BRCA1*-driven tumors have been accessed. Even though *BRCA1* breast carcinomas harbor in the majority of the cases a TN phenotype (85% of the cases), luminal (ER-positive) or HER2-positive phenotypes also have been reported [22, 28]. In this case report, *HER2* is amplified with copy number >8. Grusho *et al* analyzed in depth the number of *HER2* copies per nuclei compared to the number of centromeres of chromosome 17 [22]. None of the *BRCA1* tumors demonstrated a high level of *HER2* amplification. The level of amplification ranged around 2.4 ± 0.4. However, the co-existence of *BRCA1* constitutive mutation and *HER2* (even moderate) amplification have never been checked for the second genomic event required for the development of a hereditary tumor: *BRCA1* wild type allele inactivation. Most frequently, the wild-type allele inactivation happen through LOH (loss of chromosome or chromosome part containing the gene locus) and rarely through deleterious somatic mutation [27]. Tumor genomic profile does not display BRCAness, i.e. does not resemble the genomic profiles of TN breast tumors with homologous recombination deficiency as measured by the number of large-scale chromosomal breakpoints (LSTs) [27]. Moreover, there is no *TP53* mutation detected while the *BRCA1* breast carcinomas demonstrate a *TP53* mutation in almost all cases [29]. All these data reinforced our first hypothesis, suggesting a sporadic HER2-positive breast cancer with no hallmarks of *BRCA1*-driven tumor in a woman with *BRCA1* germline mutation.

Currently, new therapies are developed to target *BRCA1*-driven tumors. Based on the concept of synthetic lethality, PARP-inhibitors are tested in phase III trials (NCT02163694, NCT01945775, NCT02000622, NCT01905592) in women with advanced breast cancer and *BRCA* germline mutation. This case could explain the lack of response to PARP inhibitors in some of these patients. It emphasizes the importance of the genomic and molecular characterization of tumors. In women with *BRCA1* germline mutation, the incidence of “sporadic” breast cancer, with an oncogenesis independent from BRCA loss of function, is unknown but this case proves that such tumors do exist and must be taken into account when developing targeted therapies.

## MATERIALS AND METHODS

### Histopathology and immunohistochemistry

Pathological examination of the specimen was performed on haematoxylin–eosin–saffron-stained tissue-sections. Expression of ER, PR, HER2, E-cadherin, cytokeratins 5, 6, 14, 8, 18 was assessed as previously described [30]. Internal and external controls for each antibody were included in the experiments.

### Fluorescence *in situ* hybridization analysis

FISH experiments were carried out using the HER2/neu gene amplification detection system, following the instructions given by the supplier. A dual color FISH of *ERBB2* and centromere of chromosome 17 (Path-Vysion HER-2 kit, Vysis) was performed.

### TP53 sequencing

Classical Sanger sequencings were performed for *TP53* exons 4 to 10. Each PCR was performed on 30 ng of tumor DNA according to Manie et al [29]. Amplification and bi-directionally sequencing was performed using Big Dye Terminator chemistry (Applied Biosystems, Foster City, CA) with an ABI PRISM 3700 DNA Analyzer. Primer sequences are available on request.

### SNP6.0 profiling

SNP-array profiling was performed using the chip Affymetrix SNP6.0 according to the manufacturer's protocol (Affymetrix, Santa Clara, CA). Briefly, 250 ng of gDNA were digested with both Nsp and Sty restriction enzymes in independent parallel reactions, ligated to the adaptors, and amplified by PCR using a universal primer. After purification of PCR products with SNP Clean magnetic beads (Agencourt Biosciences, Beverly MA), amplicons were quantified, fragmented, labeled, and hybridized to SNP6.0 arrays.

### Analysis of genomic alterations profile

After normalization using Genotyping Console (GenomeWideSNP_6. hapmap270.na31.r1.a5) provided by Affymetrix (GTC3.0.1) SNP6 arrays were processed using the Genome Alteration Print (GAP) method to obtain absolute copy number profiles (24). Genomic BRCAness was evaluated based on the number of Large-scale State Transitions (LST) in tumor genomic profile as previously described (27). Briefly, an LST was defined as a chromosomal break (change in copy number or major allele counts) between adjacent regions of at least 10MB obtained after smoothing and filtering small-scale copy number variation (less than 3 Mb). Tumor profile is “BRCA1-like” if the number of LSTs is equal or larger than 15 (ploidy 2, near-diploid tumors) or 20 (ploidy 4, near-tetraploid tumors). Tumor ploidy was set to 2, if DNA index < 1.3, or 4 if DNA index ≥ 1.3; DNA index is average copy number calculated for tumor genome (27).

### *BRCA1* and *BRCA2* testing

DNA was extracted from a blood sample according to standard procedures [31]. The mutation screening of the *BRCA1* and *BRCA2* was performed by using the EMMA method with ad hoc primers [32]. The PCR product showing an abnormal EMMA profile was sequenced in both directions using the BigDye Terminator Cycle Sequencing V1.1 Ready Reaction kit (Applied Biosystems) followed by electrophoresis in an ABI PRISM 3130XL. *BRCA1* nucleotide position was numbered on the basis of the coding sequence NM_007294.2. The identified mutation was confirmed on a second DNA sample extracted from a buccal swab.

The *BRCA1* mutation was confirmed in the tumor DNA by direct sequencing (see above) using a couple of specific primers surrounding the mutation c.3417delT; p.Ser1139ArgfsX16 (primer sequence available on request).

## SUPPLEMENTARY MATERIALS FIGURE


