# Supplementary material for: First description of a sporadic breast cancer in a woman with BRCA1 germline mutation
Source: Oncotarget. 2015 Sep 29;6(34):35616–24. doi: 10.18632/oncotarget.5348 (PMC4742129; doi:10.18632/oncotarget.5348)
Supplement: Supplementary file 1 [file oncotarget-06-35616-s001.pdf]

SUPPLEMENTARY FIGURE

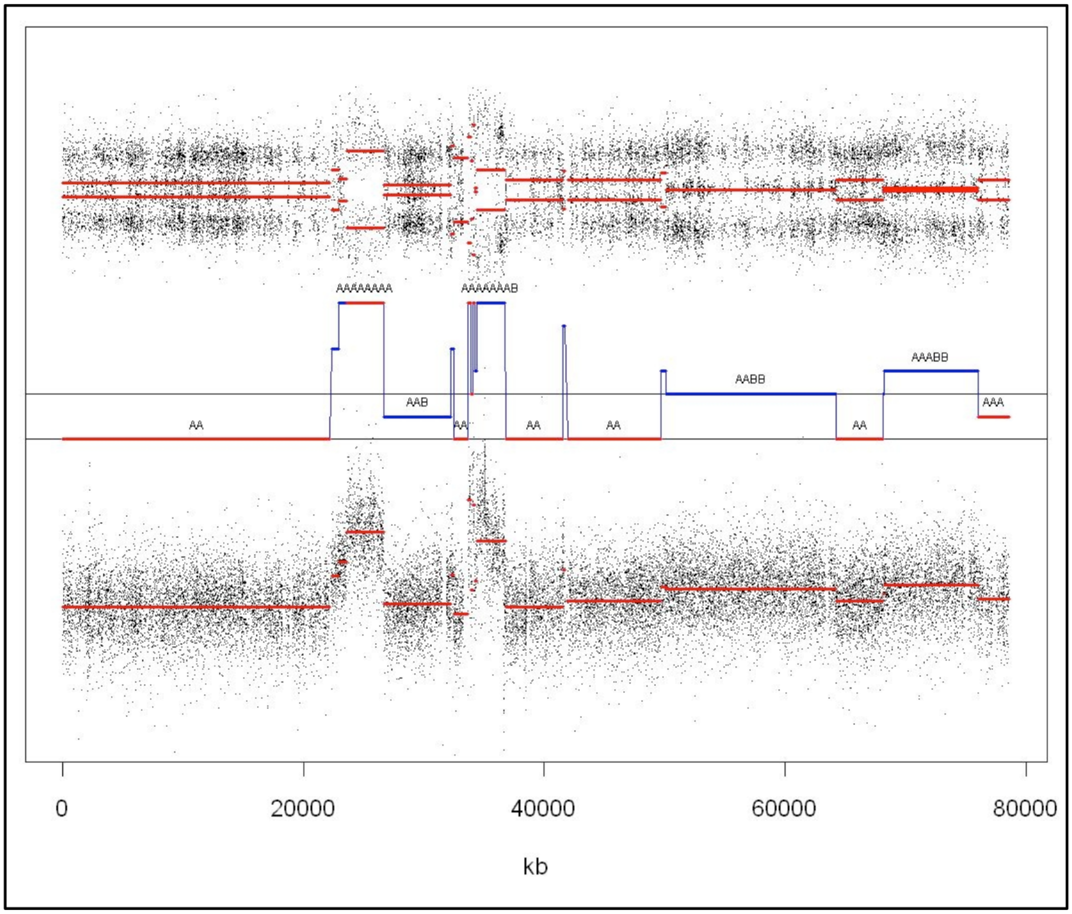

Supplementary Figure S1: Zoomed genomic profile of chromosome 17 on the *BRCA1* gene region (43,045,678-43,124,096 bp). The *BRCA1* locus has a copy number of 2 and it harbors a loss of heterozygosity (allelic status AA in a context of a tetraploid tumor). The *BRCA1* gene is not in an amplified region.
